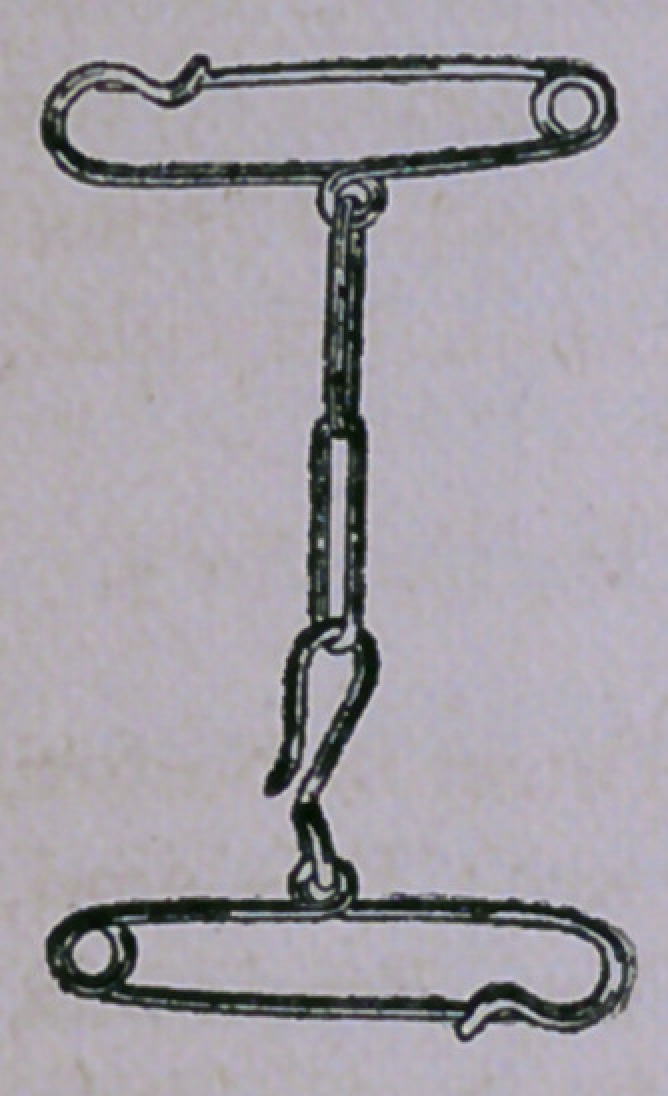# Notes and Notices

**Published:** 1895-07

**Authors:** 


### NOTES AND NOTICES.

DR. JACKSON'S OBSTETRIC AND GYNÆCOLOGICAL PINS constitute a novel device, simple, effective, and inexpensive to supply long-desired means for securing and maintaining any required position in operations in these departments. Simple in construction, small in size, of ready adjustment, and of assured aseptic possibilities, they are destined to supersede in general use the unwieldly Yoke and all other cumbersome and ineffective appliances for this purpose. They can be carried without inconvenience in pocket, bag, or case; and be, therefore, always at hand when needed, and, no special skill being required in their speedy adjustment, professional aid may, by their employment, be dispensed with in many cases. These Pins, with their attachments, are made of the finest steel, heavily plated with nickel, and are guaranteed not to break. The position may be maintained indefinitely, and no injury can result from struggles of the patient or through their use in any way. Price, one dollar per set. Obtained of Codman & Shurtleff, 13 and 15 Tremont Street, Boston, Mass.; Otis Clapp & Son, 10 Park Square, Boston, Mass.; Leach & Green, 4 Park Square, Boston, Mass.; Samuel H. Jackson, M. D., 335 Centre Street, Jamaica Plain, Mass.

The International Hahnemannian Association.—This organization had one of its enjoyable and instructive meetings at Watch Hill, Rhode Island, the scene of one of its former sessions. The attendance was good and the enthusiasm at "white heat."

The officers elected were: President, B. Fincké, M. D., of Brooklyn; Vice-President, Mary Florence Taft, M. D., Newtonville, Mass.; Secretary, Erastus E. Case, M. D., Hartford, Conn.; Corresponding Secretary, Wm. P. Wesselhœft, M. D., Boston, Mass.; Treasurer, Franklin Powel, M. D., Chester, Pa.; Censors, B. L. B. Baylies, M. D., Brooklyn, N. Y.; A. R. Morgan, M. D., Waterbury, Conn.; C. W. Butler, M. D., Montclair, N. J.; F. O. Pease, M. D., Chicago, Illinois; Alice B. Campbell, M. D., Brooklyn, N. Y.

A full report of these proceedings will appear later.

The Homœopathic Medical Society of Pennsylvania will hold its regular annual meeting at the Pittsburg Homœopathic Hospital, on the 17th, 18th, and 19th of September. The Allegheny County Homœopathic Medical Society, through its Committee of Arrangements, extend to the members of the State Society a most cordial invitation to be present. From indications the number of papers to be presented will probably exceed that of former years, and it is safe to promise that the interest and practical value of the same will certainly repay attendance. Arrangements are being made for social pleasure. The Western Pennsylvania Exposition will be one attraction, and as a special inducement the committee have projected a boat ride on the famous Monongahela River, passing through the wonderful scenery of this beautiful valley. The boat on the trip will pass the Carnegie Steel Company's plant, at Bessemer, whose wonders must be seen to be appreciated, and also touch at Homestead, a place of more than local fame. Passing up the river a number of miles, the party will have an opportunity to view the locks and dams belonging to the Monongahela Navigation Company, returning during the evening. As an accompaniment to the other pleasures of the trip, the committee will provide for the satisfying of the inner man. Particulars of the meeting will be given each member through the annual circular, which will be issued about the 1st of September.

J. Richey Horner, M. D., *Cor. Sec'y.*

The National Society of Electro-Therapeutists will meet in Boston, on September 18th and 19th, 1895. The officers for the year are: President, William L. Jackson, M. D., 685 Boylston Street, Boston; Vice-Presidents, E. S. Baily, M. D., 3034 Michigan Avenue, Chicago, Ill.; F. A. Gardner, M. D., 1016 Fourteenth Street, N. W., Washington, D. C.; Secretary, Clara

E. Gary, M. D., 546 Columbus Avenue, Boston; Treasurer, John B. Garrison, M. D., 111 East 70th Street, New York. The Executive Committee consists of the above officers and William H. King, M. D., 64 West 51st Street, New York, and M. D. Youngman, M. D., 1618 Pacific Avenue, Atlantic City, N. J.

The following are the various bureaus and their chairmen: Bureau of Electricity in Gynecology, Chairman, F. M. Frazer, M. D., 253 West 57th Street, New York. Bureau of Electricity in Diseases of the Nervous System, Chairman, E. P. Colby, M. D., 229 Berkeley Street, Boston. Bureau of General Electro-Therapeutics, Chairman, A. K. Crawford, M. D., 70 State Street, Chicago, Ill. Bureau of Electro-Surgery, Chairman, L. Willard Reading, M. D., 1629 Green Street, Philadelphia, Pa. Bureau of Electricity in Diseases of the Eye, Ear, and Throat, Chairman, Thomas L. Shearer, M. D., 345 North Charles Street, Baltimore, Md. Already many instructive and valuable papers have been promised, and the meeting bids fair to be an important one. All physicians interested in electro-therapeutics are invited to join the Society. Application for membership may be made to the Secretary. The annual dues are $2.00. A large attendance is expected, as special efforts have been made to render this meeting unusually attractive.

CLARA E. GARY, M. D., *Secretary*,
546 Columbus Avenue, Boston.

CLINICS.—Until September 1st, 1895, clinics will be held in the general amphitheatre of Cook County Hospital, Chicago, every Thursday afternoon, as follows: Medical Clinic, 2–3 P. M., conducted by Prof. F. O. Pierce, M. D. Surgical Clinic, 3–4 P. M., conducted by Prof. L. D. Rogers, M. D. Gynæcological Clinic, 4–5 P. M., conducted by Prof. Curtis M. Beebe, M. D.

These clinics are free to visitors and to practitioners residing in Cook County.

THE STANDARD DICTIONARY OF FUNK & WAGNALLS. DESCRIPTION OF THE COMPLETE INDEX.—One feature of this index is a series of notches cut into the edge of the book, with the letters of the alphabet, stamped in gold, on pieces of leather (not paper) placed in the notches exactly at the page where needed. The notches are handsomely colored, and, with the gilding on the dark pieces of leather, present a very pleasing appearance. Another, and an important feature, is the alphabetical printing on the margins of the covers and upon the margins of all pages throughout the book. This complete index enables one to turn to any letter in the book with a single motion, either when the book is shut or from *any page* at which it may be open. If the book is lying with the front cover up, and it is desired to open to any word beginning with G, pass the finger under that letter on the cover, into the notch beneath it, and the book is opened instantly to the position of the open volume. *From this position* one turns in the same way to any letter from H to M. To turn to any letter visible in the notches, as A to F, or N to Z, place the thumb on the letter in the notch, then grasp the adjacent leaves with the fingers, and the book is opened as before at a single motion.

## Figures and Tables

**Figure f1:**